# Role of S-Palmitoylation by ZDHHC13 in Mitochondrial function and Metabolism in Liver

**DOI:** 10.1038/s41598-017-02159-4

**Published:** 2017-05-19

**Authors:** Li-Fen Shen, Yi-Ju Chen, Kai-Ming Liu, Amir N. Saleem Haddad, I-Wen Song, Hsiao-Yuh Roan, Li-Ying Chen, Jeffrey J. Y. Yen, Yu-Ju Chen, Jer-Yuarn Wu, Yuan-Tsong Chen

**Affiliations:** 1 Institute of Biomedical Sciences, Academia Sinica, Taipei, Taiwan; 2 Institute of Chemistry, Academia Sinica, Taipei, Taiwan; 3 Department of Biology, University of Toronto Mississauga, Mississauga, Ontario, Canada; 4 Department of Pediatrics, Duke University Medical Center, Durham, North Carolina United States of America

## Abstract

Palmitoyltransferase (PAT) catalyses protein S-palmitoylation which adds 16-carbon palmitate to specific cysteines and contributes to various biological functions. We previously reported that in mice, deficiency of *Zdhhc13*, a member of the PAT family, causes severe phenotypes including amyloidosis, alopecia, and osteoporosis. Here, we show that *Zdhhc13* deficiency results in abnormal liver function, lipid abnormalities, and hypermetabolism. To elucidate the molecular mechanisms underlying these disease phenotypes, we applied a site-specific quantitative approach integrating an alkylating resin-assisted capture and mass spectrometry-based label-free strategy for studying the liver S-palmitoylome. We identified 2,190 S-palmitoylated peptides corresponding to 883 S-palmitoylated proteins. After normalization using the membrane proteome with TMT10-plex labelling, 400 (31%) of S-palmitoylation sites on 254 proteins were down-regulated in *Zdhhc13*-deficient mice, representing potential ZDHHC13 substrates. Among these, lipid metabolism and mitochondrial dysfunction proteins were overrepresented. MCAT and CTNND1 were confirmed to be specific ZDHHC13 substrates. Furthermore, we found impaired mitochondrial function in hepatocytes of *Zdhhc13*-deficient mice and *Zdhhc13*-knockdown Hep1–6 cells. These results indicate that ZDHHC13 is an important regulator of mitochondrial activity. Collectively, our study allows for a systematic view of S-palmitoylation for identification of ZDHHC13 substrates and demonstrates the role of ZDHHC13 in mitochondrial function and metabolism in liver.

## Introduction

S-Palmitoylation is a post-translational modification whereby a palmitate group is covalently attached to a specific cysteine residue via a thioester linkage. This modification enhances protein hydrophobicity, affects protein membrane association, and alters protein subcellular localization. In addition, palmitoylation plays important roles in protein functions, such as protein trafficking, protein targeting, protein stability, and protein–protein interactions. Notably, palmitoylation is reversible, allowing for dynamic regulation of protein function and participation in diverse aspects of cellular signalling^1–3^. Therefore, it is important to gain a deeper understanding of the underlying mechanisms and regulation of protein palmitoylation.

Palmitoylation is catalysed by palmitoyltransferases (PATs). In mammals, 23 ZDHHC genes encode PATs with aspartate-histidine-histidine-cysteine (DHHC) motif^4–6^; the cysteine residue in this motif is critical for PAT enzyme activity. Although individual PATs display substrate specificity, some substrates can be modified by more than one PAT^7, 8^. For instance, huntingtin is palmitoylated by ZDHHC17 and ZDHHC13^9^, and SNAP-25 is palmitoylated by ZDHHC2, ZDHHC3, ZDHHC7, ZDHHC15, and ZDHHC17^10^.

Recent advances in mass spectrometry (MS)-based proteomics and the development of techniques that can selectively enrich palmitoylated proteins have enabled the high-throughput characterization of S-palmitoylation. Acyl-biotin exchange (ABE) is widely used in studies of the palmitoylated proteome^11–14^. However, despite the potential of this technique for large-scale identification of palmitoylated proteins in different biological systems, a large number of initial samples is required owing to protein loss during the complicated methodological procedures. Resin-assisted capture (RAC) has been used in lieu of ABE to purify S-acylated and palmitoylated proteins (acyl-RAC)^15^. Similar to ABE, the acyl-RAC method also involves blocking free thiols with methyl methanethiosulfonate (MMTS), cleavage of thioester linkages between cysteine and palmitate, and capture of nascent thiols on thiopropyl Sepharose. Compared to ABE, this strategy is simpler and the captured peptides are more acceptable to MS owing to the absence of biotin labelling; hence, this method has increased the sensitivity for the identification of palmitoylated peptides. However, palmitoylation sites are difficult to determine unambiguously when a peptide contains two or more cysteine residues. Metabolic labelling with a palmitic acid analog followed by click chemistry (MLCC) has been applied in palmitoylated protein research^16, 17^. Nevertheless, MLCC has been used in mammalian cells rather than in live animals, and only capture proteins in the metabolic labelling time course.

To date, palmitoylation has been investigated partly because PATs have been linked to several human conditions, including Huntington’s disease (ZDHHC13 and ZDHHC17)^18, 19^, schizophrenia (ZDHHC8)^20^, type 1 diabetes (ZDHHC17)^21^, intellectual disability (ZDHHC9 and ZDHHC15)^8, 22^, and multiple types of cancers (ZDHHC2, ZDHHC9, ZDHHC11, ZDHHC14, and ZDHHC20)^23^. Furthermore, mice with an F233 deletion in ZDHHC21 show abnormalities in skin homeostasis and hair defects^24^. To understand the function of disease-associated PATs, it is critical to link enzymes to their corresponding substrates. To date, only six proteomics studies have focused on identifying substrates of specific PATs, such as ZDHHC2, ZDHHC5, and ZDHHC17^25–28^. We previously reported that ZDHHC13 is a novel regulator of postnatal skeletal development and bone mass acquisition via palmitoylation of MT1-MMP^29^. Moreover, ZDHHC13 is important for hair anchoring and skin barrier functions, likely mediated by the ZDHHC13 substrate, cornifelin^30^. Previously, we generated a *Zdhhc13*-deficient mouse model through N-ethyl-N-nitrosourea (ENU) mutagenesis^31^. The mutation creates a truncated protein (R425X) that lacks the zinc-finger DHHC-CRD domain (positions 426–476) and the active site (C456) for the formation of an S-palmitoyl cysteine, resulting in nonsense-mediated mRNA decay of *Zdhhc13* mRNA. Compared with other PAT family member-defective mouse models, *Zdhhc13-*deficient mice exhibit severe phenotypes, including alopecia, osteoporosis, and amyloidosis. In this study, we aimed to investigate the metabolic abnormalities, including hypermetabolism, and defective lipid metabolism of *Zdhhc13-*deficient mice. Furthermore, given that few ZDHHC13 substrates have yet been identified, we studied the liver S-palmitoylome using a quantitative proteomics approach that combined the alkylating RAC method and an MS-based label-free strategy to identify candidate ZDHHC13 substrates.

## Results

### Abnormal liver function, lipid abnormalities, and hypermetabolism in *Zdhhc13*-deficient mice

**Figure 1:**
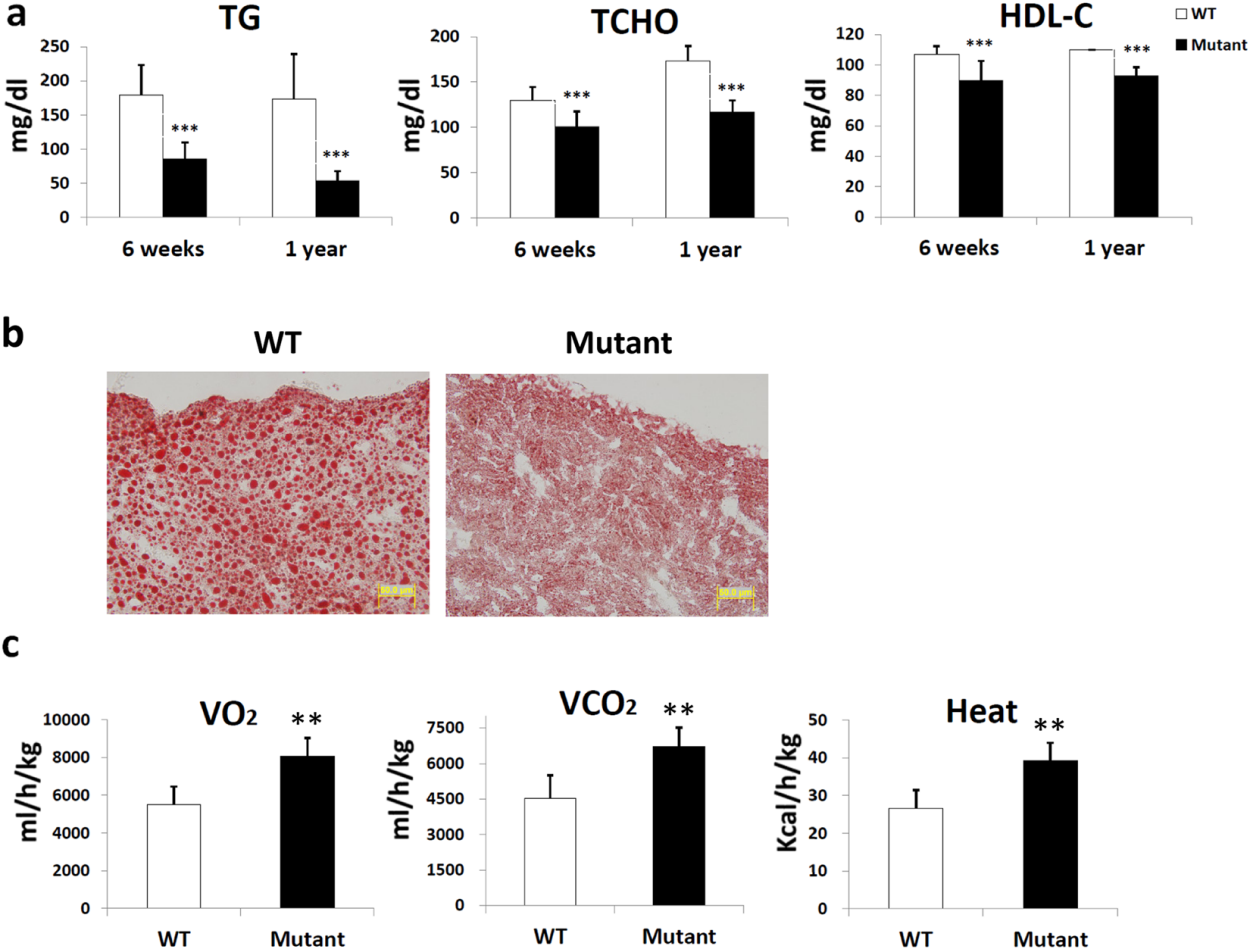
Metabolic abnormalities in *Zdhhc13*-deficient mice. (**a**) The serum level of TG/TCHO/HDL-C in *Zdhhc13*-deficient mice at the ages of 6 weeks and 1 year (n = 12). (**b**) Lipid accumulations in liver section were examined with Oil Red O stain at the age of 1 year (n = 3). (**c**) O_2_ consumption and CO_2_ and heat production in *Zdhhc13*-deficient mice and WT mice (n = 8). Triglyceride (TG; mg/dl), total cholesterol (TCHO; mg/dl), high-density lipoprotein cholesterol (HDL-C; mg/dl). *P < 0.05, **P < 0.005, ***P < 0.001.

In addition to our previous report that *Zdhhc13*-deficient mice presented with osteoporosis, alopecia, skin abnormalities, and amyloidosis^31^, the mice also exhibited elevated liver transaminase. In this work, we found that these mice had low blood triglyceride and cholesterol levels, as well as decreased lipid accumulation in the liver (Fig. 1a,b). We further observed that these mutant mice displayed excessive sweating and poor weight gain despite adequate water and food intake. Therefore, we examined the metabolic state and efficiency of *Zdhhc13*-deficient mice and found that VO_2_ consumption, VCO_2_ production, and heat production were significantly increased in the mutant mice compared to those of wild-type (WT) mice (Fig. 1c). However, serum levels of T4 and T3 were not significantly different in WT and mutant mice (T3: WT: 1.05 ± 0.24 ng/ml, mutant mice: 1.27 ± 0.32 ng/ml; T4: WT: 4.6 ± 0.43 ng/ml, mutant mice: 4.3 ± 0.17 ng/ml), suggesting that this hypermetabolism was not due to hyperthyroidism.

### Identification and characterization of the liver S-palmitoylome

**Figure 2:**
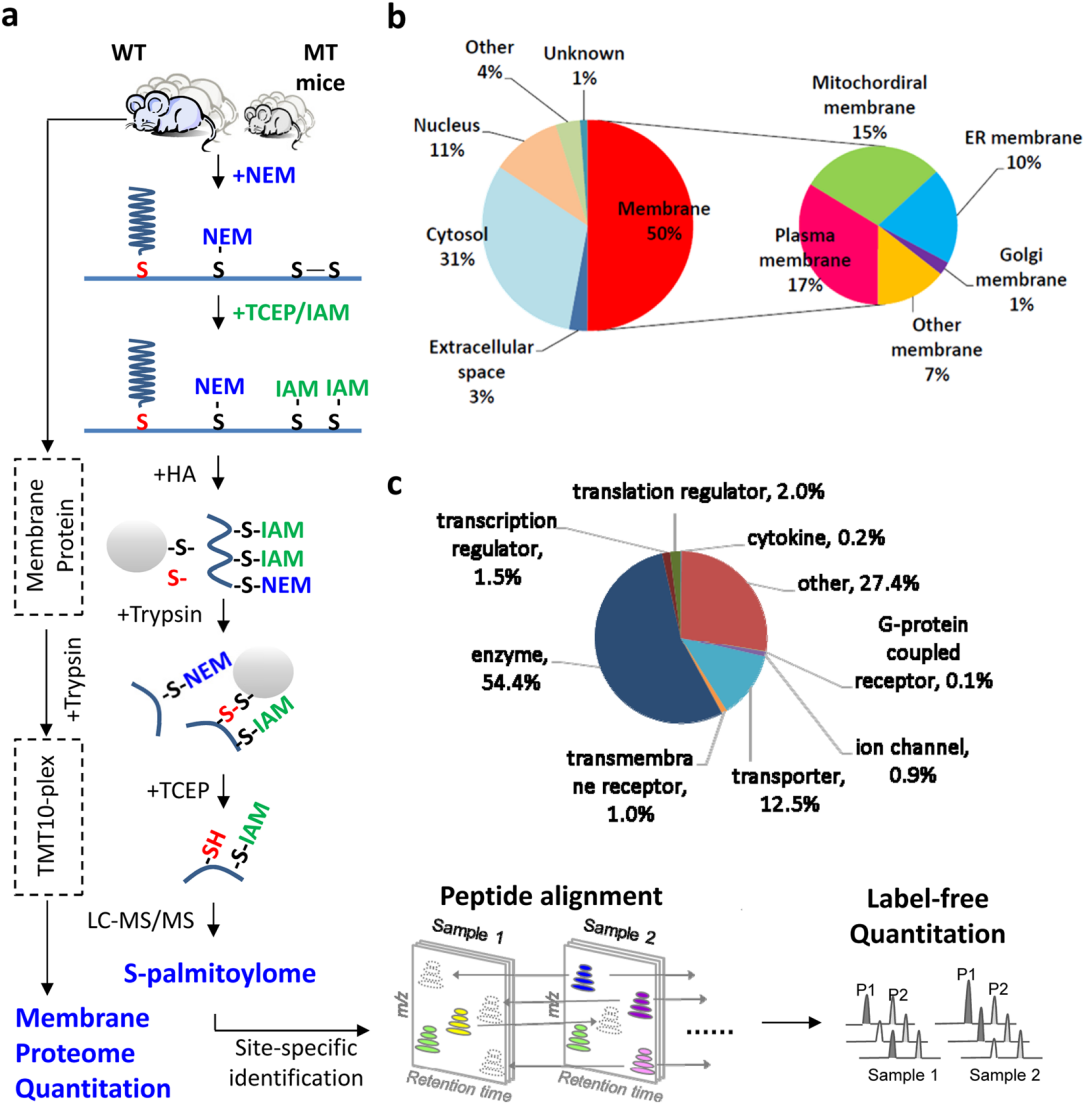
Identification and characterization of liver S-palmitoylome. (**a**) A schematic overview for the site-specific identification of the S-palmitoylome by a modified alkylating RAC assay. Free thiols are first blocked with NEM. Disulfide bond was cleaved by TCEP and further blocked with IAM. Thioesters are then cleaved with neutral HA and the newly liberated thiols are purified with thiol sepharose 4B. The capture proteins on sepharoses were digested with trypsin. The supernatant of digested samples was removed and the captured peptides on sepharoses were eluted with TCEP and subjected to downstream MS analysis for S-palmitoylated site identification. Peptide alignment was applied to increase the coverage based on the retention time and m/z of identified peptides. Extracted ion chromatogram of identified peptides and the same m/z in different LC samples were used to quantify the relative abundance. The protein levels were quantified by TMT10-plex labelling. (**b**) Pie diagram illustrating the subcellular localization of identified S-palmitoylated proteins. (**c**) Pie diagram illustrating the functions of identified S-palmitoylated proteins. NEM, N-ethylmaleimide; IAM, Iodoacetamide; HA, hydroxylamine.

To explore the pathological mechanisms underlying hypermetabolism and defective lipid metabolism in *Zdhhc13-*deficient mice, we analysed tissue samples from the liver, a key metabolic organ. For site-specific identification and quantitation of the S-palmitoylome and systematic delineation of S-palmitoylated substrates of ZDHHC13 from WT and *Zdhhc13-*deficient mice, we used a simple multiplex strategy that combines an alkylating RAC method and an informatics-assisted label-free strategy^32^ (Fig. 2a). Using alkylating agents for blocking free and disulfide cysteines, the false positive signals that originate from protein or reagent disulfide exchange can be minimized. With the covalent tagging of these cysteines, S-palmitoyl groups on proteins were reduced by hydroxylamine, generating a reducing form of the cysteine residue. Followed by thiol-resin capture and eluted by TCEP, unambiguous palmitoylation sites can be specifically identified as reduced cysteine residues. To further increase identification coverage and decrease quantitation of heterogeneity from individual mice, we also utilized peptide alignment to discover signals of un-identified S-palmitoylated peptides in different LC-MS/MS replicates based on the corresponding retention time and m/z of identified peptides. Finally, all S-palmitoylated peptides were relatively quantified with extracted ion chromatogram (XIC). In addition, expression levels of membrane proteins were quantified by TMT10-plex labelling for normalization.

In total, 2,190 unique S-palmitoylated peptides (1,905 sites) corresponding to 883 proteins were identified from three pairs of WT and *Zdhhc13-*deficient mice (mascot score ≥25, p < 0.05, Table S1), including well-known palmitoylated proteins such as CD82, CPS1, Gnas, Rras, Gphn, TMX, Ctnnd2 and M6pr. Among these, 311 (35%) were novel S-palmitoylated proteins that had not been previously identified (Table S2). These results demonstrate that alkylating RAC combined with peptide alignment of the liver S-palmitoylome provides greater sensitivity for identification of palmitoylated proteins than that of the previously used ABE system. To explore the potential functional roles of these 883 S-palmitoylated proteins in mice, subcellular localizations and functions were annotated using the Ingenuity Pathway Analysis (IPA) Knowledge Base and the Gene Ontology (GO) consortium. As shown in Fig. 2b, 50% of identified S-palmitoylated proteins are located in the membrane fraction, including the plasma membrane (17%), mitochondrial membrane (15%), endoplasmic reticulum (10%), Golgi apparatus (1%), and other membrane (7%). In addition, 31% of S-palmitoylated proteins are located in the cytosol, 11% in the nucleus, and 3% in the extracellular space. The functional categorization of these S-palmitoylated proteins revealed that most proteins are enzymes (54.4%) and many are involved in metabolism (Fig. 2c). In addition, some S-palmitoylated proteins were categorized as transporters (12.5%), translation regulators (2%), transcription regulators (1.5%), and transmembrane receptors (1%).

### Identification and characterization of candidate ZDHHC13 substrates

Candidate ZDHHC13 substrates were identified as S-palmitoylated peptides that were significantly down-regulated (≤0.5-fold) in triplicate samples of *Zdhhc13-*deficient mice vs. WT mice. In order to remove the effects of changes in protein expression on S-palmitoylation levels, we used the corresponding protein level of each protein to normalize the S-palmitoylation levels. Following quantitation of protein levels with TMT10-plex labelling, expression levels of approximately 96% of proteins (3,117 of 3,246) were unchanged, with fold-changes between 0.71 and 1.41 (average ratio = 1.06 ± 0.34 with 95% confidence). Based on the biological functions and subcellular localizations, some outlier proteins as well as general contaminant proteins from immunoprecipitation or affinity capture (such as ribosomal proteins, refer to CRAPome database: http://www.crapome.org/) were filtered. The remaining 254 proteins corresponding to 400 S-palmitoylation sites (369 peptides) were annotated as potential ZDHHC13 substrates (Table S4).

**Figure 3:**
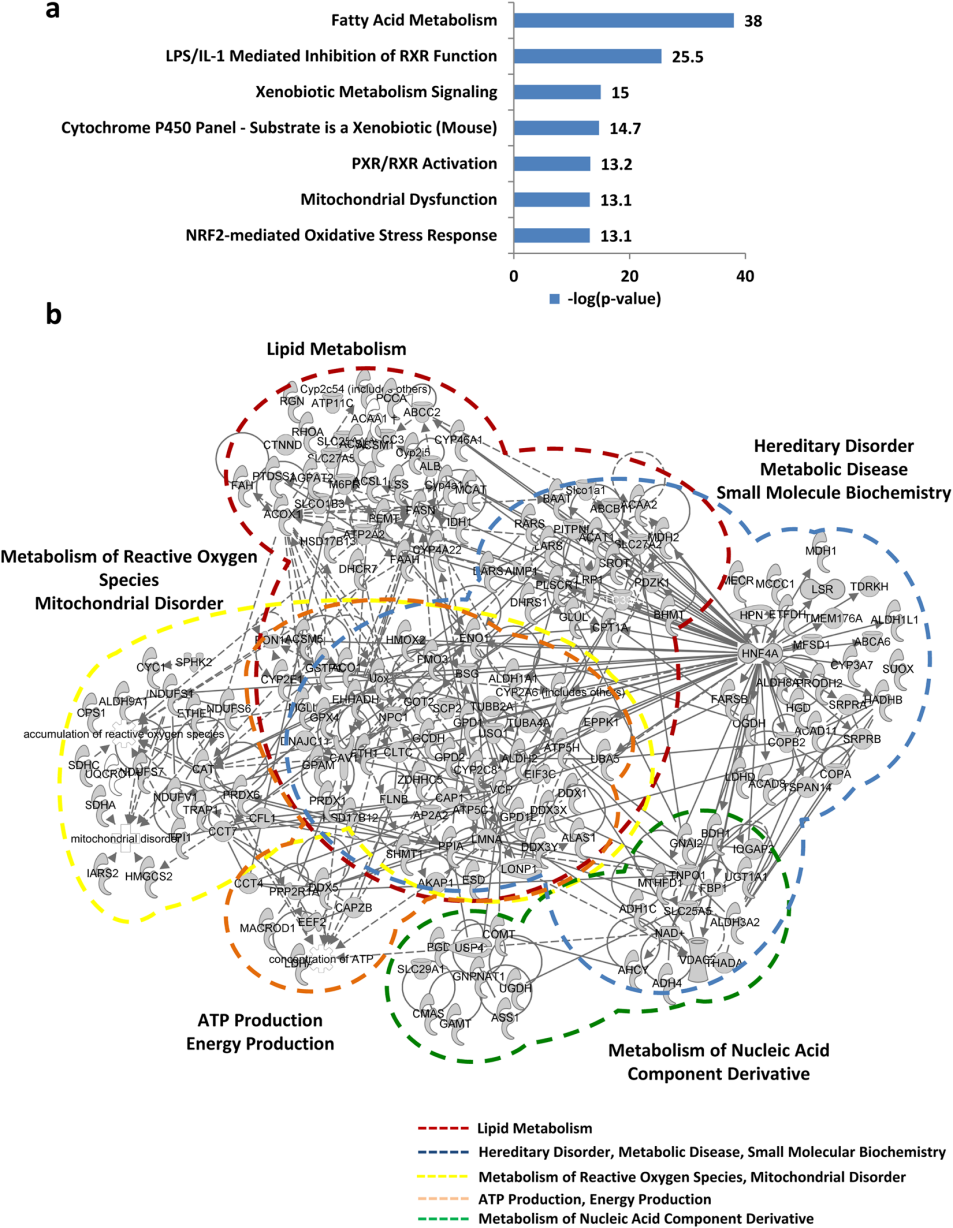
Functional network of candidate ZDHHC13 substrates. (**a**) Bar chart illustrating the toxicity function of identified candidate ZDHHC13 substrates. (**b**) Systematic network presenting the functions of candidate ZDHHC13 substrates in mitochondrial dysfunction, lipid metabolism, metabolic disease, and other correlated functions.

We further studied the canonical pathway and functional annotations by IPA and found that many of these proteins are associated with liver-related disorders and metabolism, especially fatty acid metabolism and mitochondrial dysfunction (Table 1, Fig. 3a,b). Of these proteins, 46% are involved in metabolism, including MCAT, SDHA, NDUFV1, NDUFS1, FASN, ACAA1, and ACAA2 (Table S5). A systematic network of protein–protein interactions showed that these candidate ZDHHC13 substrates are associated with energy production, metabolic diseases, and the maintenance of cellular function (Fig. 3b). In addition, we identified many enzymes involved in lipid metabolism. In order to eliminate the possibility that these enzymes form covalent acyl-enzyme intermediates during catalysis, we confirmed the palmitoylation of three proteins, ACAA2, MCAT, and CTNND1, by MLCC and verified that they are indeed palmitoylated proteins (Fig. S1). Taken together, the quantitative study of the S-palmitoylome indicated that ZDHHC13 and S-palmitoylated proteins that are down-regulated in *Zdhhc13*-deficient mice play important roles in metabolism and mitochondrial dysfunction.

**Table 1:** Partial list of candidate ZDHHC13 substrates involved in mitochondrial dysfunction and lipid metabolism.

UniProt	Protein	Gene	m/z	Charge	Modified peptide	Palm site	Palm/proteome	Palm level K+/WT+(ave)	Proteome level K/WT (ave)
Q8R3F5	Malonyl CoA-acyl carrier protein transacylase, mitochondrial	Mcat	703.9	2	VLGYDLLEL**c**LR	104	0.325	0.314	0.965
P30999	Catenin delta-1	Ctnnd1	774.0	3	YQEALPTVANSTGPHAAS**c**FGAK	618	0.466	0.501	1.074
			914.9	2	HIEWESVLTNTAG**c**LR	533	0.465	0.500	1.074
Q8K2B3	Succinate dehydrogenase [ubiquinone] flavoprotein subunit, mitochondrial	Sdha	850.9	2	AAFGLSEAGFNTA**c**LTK	89	0.340	0.388	1.141
			760.9	2	A**c**ALSIAES**c**RPGDK	467,475	0.435	0.496	1.141
			785.9	2	TLNEAD**c**ATVPPAIR	654	0.433	0.494	1.141
			574.8	2	VGSVLQEG**c**EK	536	0.224	0.256	1.141
Q91YT0	NADH dehydrogenase [ubiquinone] flavoprotein 1, mitochondrial	Ndufv1	1095.9	3	LKPPFPADVGVFG**c**PTTVANVETVAVSPTI**c**R	238,255	0.431	0.490	1.136
			853.9	2	NA**c**GSDYDFDVFVVR	187	0.460	0.523	1.136
			802.4	3	QIEGHTI**c**ALGDGAAWPVQGLIR	425	0.412	0.468	1.136
			622.6	3	YLVVNADEGEPGT**c**KDR	125	0.487	0.554	1.136
Q91VD9	NADH-ubiquinone oxidoreductase 75 kDa subunit, mitochondrial	Ndufs1	752.4	2	AVTEGAQAVEEPSI**c**	727	0.491	0.560	1.141
			482.7	2	M**c**LVEIEK	78	0.463	0.528	1.141
Q9Z1P6	NADH dehydrogenase [ubiquinone] 1 alpha subcomplex sub	Ndufa7	567.3	2	LSNNYY**c**TR	55	0.363	0.412	1.134
Q9CZ13	Cytochrome b-c1 complex subunit 1, mitochondrial	Uqcrc1	648.8	2	L**c**TSATESEVTR	380	0.264	0.300	1.133
			774.9	2	VYEEDAVPGLTP**c**R	268	0.481	0.545	1.133
Q8C196	Carbamoyl-phosphate synthase [ammonia], mitochondrial	Cps1	863.8	3	AERPDGLILGMGGQTALN**c**GVELFK	516	0.078	0.078	1.003
			672.9	2	GNDVLVIE**c**NLR	1256	0.351	0.352	1.003
			700.9	2	GNPTKVVAVD**c**GIK	225	−999	−999	1.003
			673.8	2	M**c**HPSVDGFTPR	816	0.494	0.496	1.003
			449.5	3	M**c**HPSVDGFTPR	816	0.305	0.306	1.003
			452.2	2	VVAVD**c**GIK	225	0.470	0.472	1.003
			452.2	2	VVAVD**c**GIK	225	0.380	0.381	1.003
Q8VCH0	3-ketoacyl-CoA thiolase B, peroxisomal	Acaa1b	882.4	2	D**c**LIPmGITSENVAER	177	−999	−999	0.837
			648.7	3	G**c**FHAEIVPVTTTVLNDK	218	−999	−999	0.837
			548.9	3	Q**c**SSGLQAVANIAGGIR	123	−999	−999	0.837
Q8BWT1	3-ketoacyl-CoA thiolase, mitochondrial	Acaa2	1098.5	2	L**c**GSGFQSIVSGCQEICSK	92	0.279	0.248	0.890
P19096	Fatty acid synthase	Fasn	834.9	2	DPETLLGYSMVG**c**QR	135	0.399	0.369	0.925
			575.3	2	LGMLSPDGT**c**R	212	0.475	0.439	0.925
			1106.9	3	STLATSSSQPVWLTAMDCPTSGVVGLVN**c**LR	1452	−999	−999	0.925
Q8QZT1	Acetyl-CoA acetyltransferase, mitochondrial	Acat1	936.5	2	QATLGAGLPISTP**c**TTVNK	116	0.453	0.428	0.944
P41216	Long-chain-fatty-acid–CoA ligase 1	Acsl1	554.0	3	EVAELAE**c**IGSGLIQK	133	0.387	0.302	0.780
			660.0	3	GAmITHQNIIND**c**SGFIK	298	−999	−999	0.780
			545.3	2	K**c**GVEIISLK	242	0.443	0.345	0.780
Q91VA0	Acyl-coenzyme A synthetase ACSM1, mitochondrial	Acsm1	786.7	3	AIVTTASLVPEVESVASE**c**PDLK	175	−999	−999	0.891
			788.4	2	VPEWWLVTVG**c**MR	127	0.490	0.437	0.891
O35488	Very long-chain acyl-CoA synthetase	Slc27a2	459.3	2	GEVGLLV**c**K	427	0.478	0.504	1.055
			734.4	2	LG**c**PMA**c**LNYNIR	129,133	0.434	0.458	1.055
			724.8	2	YL**c**NTPQKPNDR	320	0.283	0.299	1.055
			724.9	2	YL**c**NTPQKPNDR	320	0.329	0.347	1.055
			483.6	3	YL**c**NTPQKPNDR	320	0.240	0.254	1.055

Candidate ZDHHC13 substrates are list in line included the uniport number, protein name, gene name, mass to charge ratio (m/z), peptide charge, peptide sequence which the modification cysteine is shown in lower case letter, S-palmitoylated modification site, Palm/proteome, Palm level (“+” indicated the sample treated with Hydroxylamine), Proteome level. (K indicates *Zdhhc13*-deficient mice).

### Decreased palmitoylation of candidate ZDHHC13 substrates in *Zdhhc13*-deficient mice

**Figure 4:**
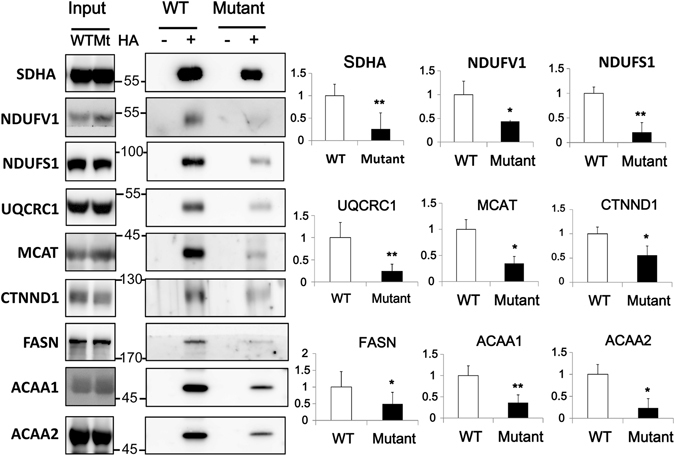
Palmitoylation levels of candidate ZDHHC13 substrates in the livers of WT and mutant mice. Liver tissue was blocked with NEM, treated with or without hydroxylamine (HA), and then the alkylating RAC method was used to purify palmitoylated proteins. The eluted proteins were analysed by immunoblotting. Endogenous protein level was used as input control. Quantitative fold changes in the WT+HA group versus the mutant +HA group are shown (right). N = 3, *P < *0.05*, **P < *0.005*.

We chose nine candidate ZDHHC13 substrates based on their functions in mitochondria and lipid metabolism, as well as their potential to cause metabolic abnormalities, to further validate S-palmitoylation levels using western blot analysis. SDHA, NDUFV1, NDUFS1, and UQCRC1, are involved in regulating the electron transport chain in mitochondria, while MCAT is involved in mitochondrial fatty acid synthesis, and FASN, ACAA1, ACAA2, and CTNND1 are involved in fatty acid synthesis and β-oxidation. As shown in Fig. 4, these proteins were significantly less palmitoylated in *Zdhhc13-*deficient mice compared with those of WT mice. These results are consistent with the proteomics data and suggest that reduced palmitoylation of candidate ZDHHC13 substrates may cause abnormal phenotypes in *Zdhhc13-*deficient mice.

### Confirmation of MCAT and CTNND1 as ZDHHC13 palmitoylation substrates

**Figure 5:**
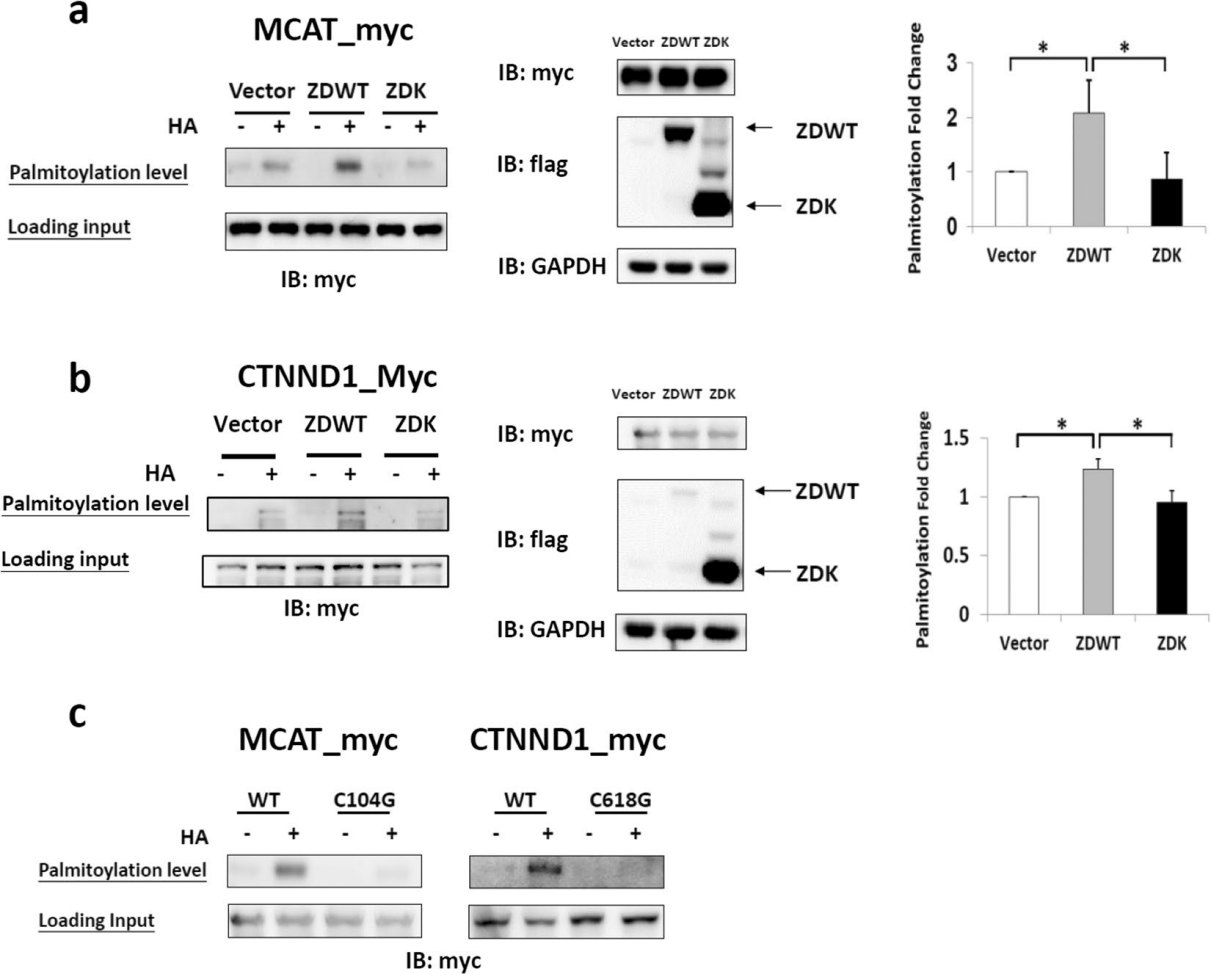
Palmitoylation levels and sites of ZDHHC13 substrates, MCAT and CTNND1. The palmitoylation level and sites of MCAT and CTNND1 were confirmed by the alkylating RAC method. (**a,b**) HEK293T cells were co-transfected with MCAT-myc or CTNND1-myc and flag vector (Vector, group 1), ZDHHC13 flag (ZDWT, group 2), or mutant ZDHHC13-flag (ZDK, group 3) for 24 h. The palmitoylation level was detected with the anti-myc antibody (left panel). Samples before thiol sepharose 4B purification were used as the loading controls. The palmitoylation level from three independent repeats is shown as a fold change to vector group (right panel). The expression levels of the substrate and ZDHHC13 were detected with anti-myc and anti-flag (middle panel). (**c**) Validation of the MS data by transfection of HEK293T cells with Mcat-myc or CTNND1-myc or their palmitoylated mutant form (MCAT-C104G, CTNND1-C618G). Sample before thiol sepharose 4B purification was examined by anti-myc as loading control. This experiment was repeated 3 times. *P < *0.05*.

We further chose two of the above proteins to confirm that they are indeed ZDHHC13 substrates. Previous studies have shown that *Mcat*-knockout mice exhibited similar phenotypes to those of *Zdhhc13-*deficient mice, including kyphosis and alopecia^33^. Here, we showed that palmitoylation levels of MCAT were significantly (2-fold) higher in ZDWT cells than basal palmitoylation levels in ZDK cells (Fig. 5a). CTNND1, a known anti-adipogenic protein^34^, has been identified as a palmitoylated protein in the lipid rafts of human prostate cancer cells in a proteomics study^35^. The S-palmitoylation level of CTNND1 was 1.25-fold higher in ZDWT co-expressing cells than in ZDK cells (Fig. 5b). These results confirm that MCAT and CTNND1 are ZDHHC13 substrates.

Our proteomics results identified C104 and C618 as palmitoylation sites in MCAT and CTNND1, respectively (Table 1). To further examine the fidelity of MS-based identification of palmitoylation sites, we generated two mutants, MCAT C104G and CTNND1 C618G, in which the cysteine residues were replaced with glycine. Reduced palmitoylation levels were observed for both mutant proteins compared with those of WT proteins (Fig. 5c). These findings not only verify that C104 of MCAT and C618 of CTNND1 are ZDHHC13 palmitoylation sites, but also confirm the fidelity of the alkylating RAC method in detecting novel palmitoylation sites.

### Impaired mitochondrial function in hepatocytes of *Zdhhc13*-deficient mice and *Zdhhc13*-knockdown Hep1-6 cells

**Figure 6:**
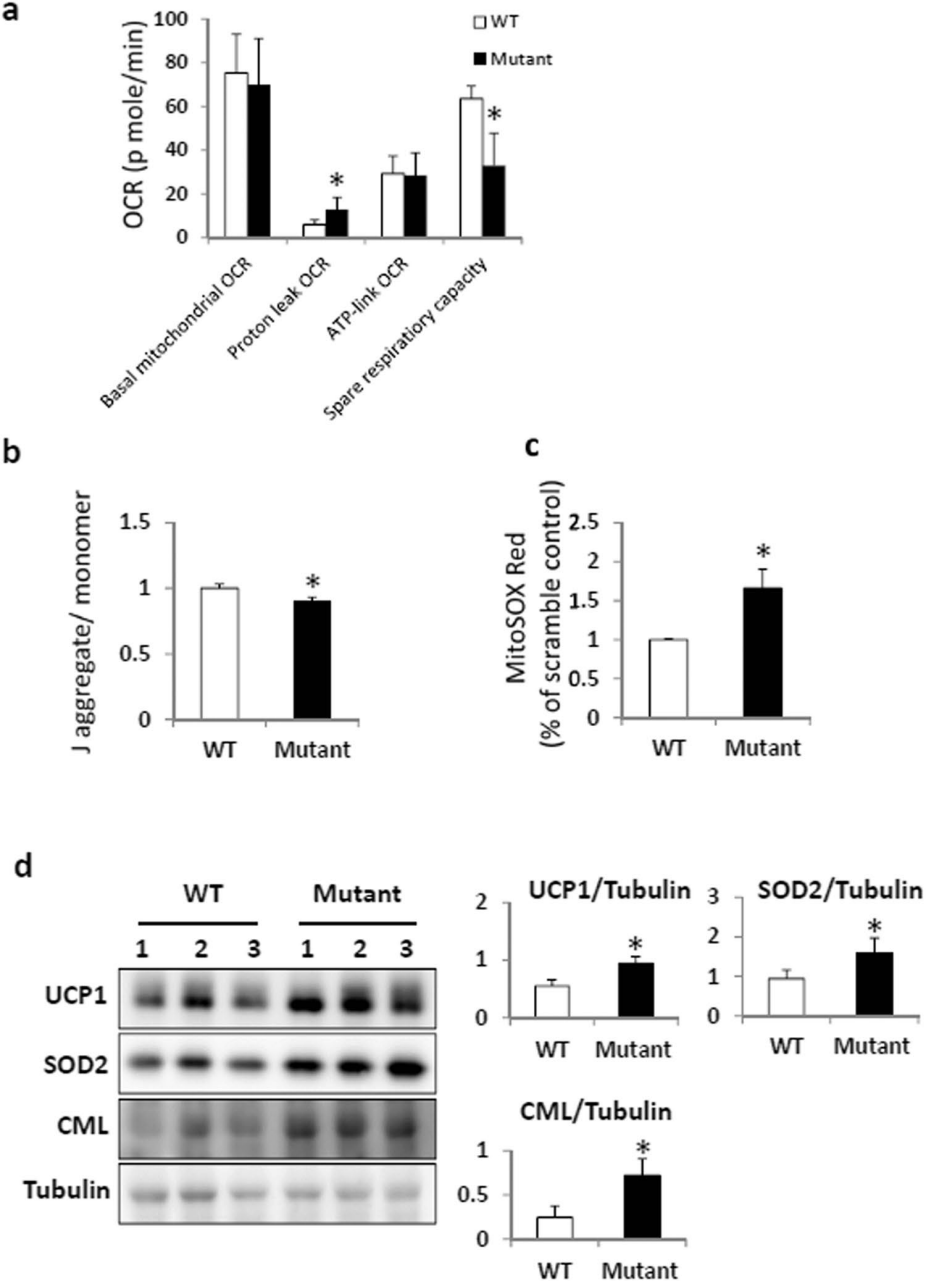
Mitochondrial function and oxidative stress in *Zdhhc13*-deficient hepatocyte. Primary hepatocytes isolated from WT and *Zdhhc13*-deficient mice were assay. (**a**) O_2_ consumption rate (OCR) of primary hepatocytes isolated from WT and *Zdhhc13*-deficient mice were examined by the Seahorse XF96 extracellular flux analysers. (**b**) Mitochondrial membrane potential in WT and *Zdhhc13*-deficient hepatocyte. JC-1 was used to detect the mitochondrial membrane potential in this experiment. (**c**) Mitochondrial ROS levels of WT and *Zdhhc13*-deficient hepatocyte. MitoSOX Red was used to assay the mitochondrial ROS level. (**d**) The protein expression patterns of UCP1, SOD2 and CML were detected by western blotting in WT and *Zdhhc13*-deficient mice hepatocyte. Tubulin was used as an internal control. Quantitative fold changes in the WT versus the *Zdhhc13*-deficient mice are shown (right). N = 6, *P < *0.05*.

**Figure 7:**
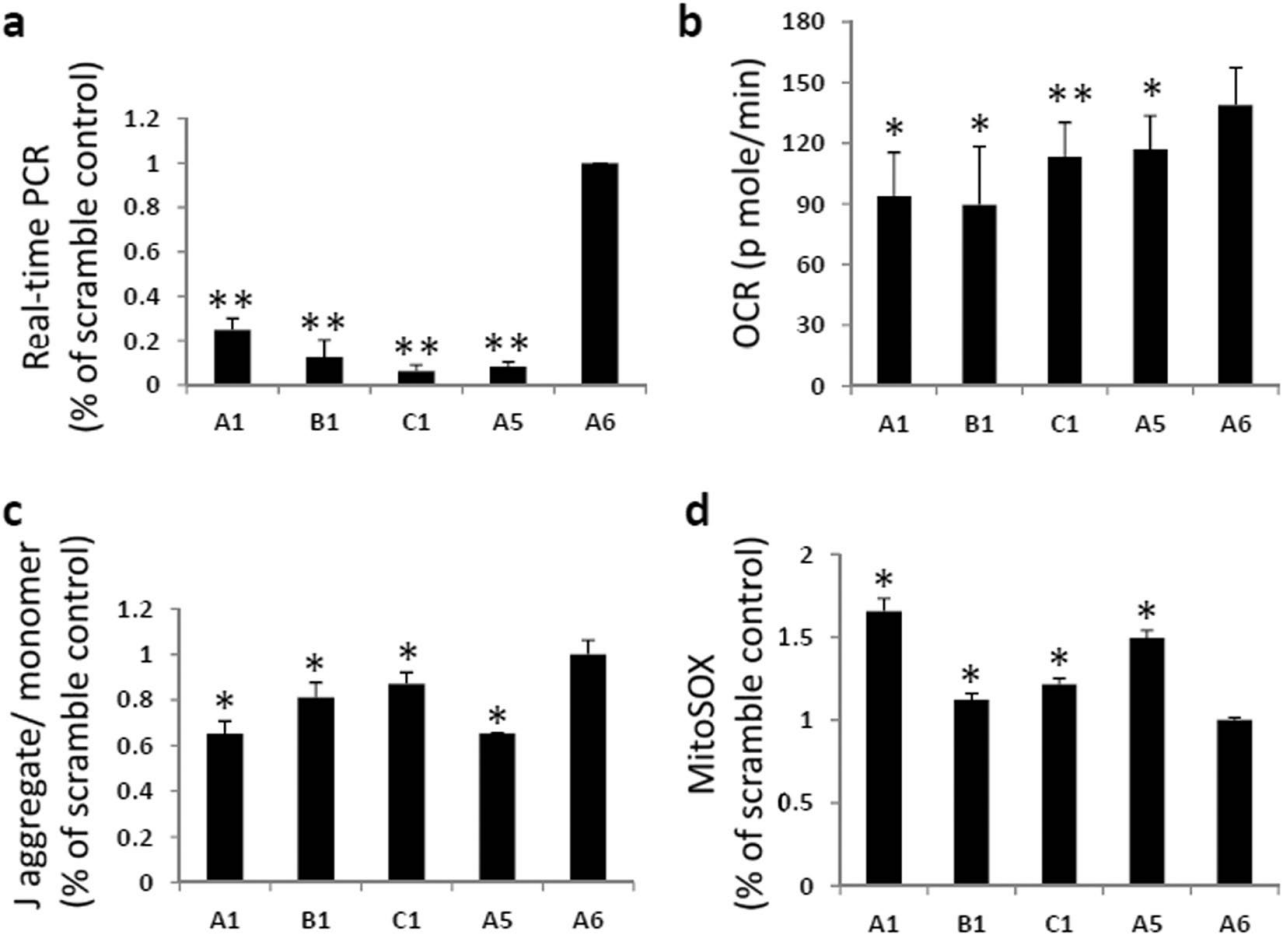
Mitochondrial function and oxidative stress in *Zdhhc13*-knock-down Hep1–6 cell. (**a**) Expression levels of *Zdhhc13* mRNA in knock-down Hep1–6 cells as examined by real-time PCR. There were 4 shRNA targeting to *Zdhhc13* (A1, B1, C1, A5), and 1 shRNA was used as scramble control (A6). (**b**) Mitochondrial membrane potential in *Zdhhc13* knock-down and scramble control cells. JC-1 was used to detect the mitochondrial membrane potential in this experiment. (**c**) Mitochondrial ROS levels of *Zdhhc13* knock-down cells and scramble controls. MitoSOX Red was used to assay the mitochondrial ROS level. (**d**) The spare respiratory capacity of *Zdhhc13* knock-down cells and scramble controls. These experiments were repeated at 3 times, *P < *0.05*., **P < *0.005*.

The systematic proteomics study of ZDHHC13 identified several candidates for S-palmitoylated substrates involved in the regulation of mitochondrial function (Fig. 3); therefore, these proteins may result in impaired mitochondrial function in *Zdhhc13*-deficient mice. To evaluate the effect of ZDHHC13 on mitochondrial function, primary hepatocytes isolated from WT and *Zdhhc13*-deficient mice were assay by the Seahorse XF96 extracellular flux analysers. The results showed that the proton-leak OCR was slightly higher in *Zdhhc13*-deficient hepatocytes than in WT hepatocytes, and the spare respiratory capacity in the presence of 4 μM FCCP was higher in *Zdhhc13*-deficient hepatocytes than in WT hepatocytes. The basal mitochondrial OCR and ATP-link OCR did not show significant difference (Fig. 6a). We further examined the mitochondrial membrane potential, mitochondrial ROS level, and oxidative damage biomarkers. The membrane potential was assessed by measuring changes in the JC-1 aggregate to JC-1 monomer ratio. The results showed that the membrane potential were slightly lower in *Zdhhc13*-deficient hepatocytes than in WT hepatocytes (Fig. 6b). The mitochondrial ROS level was assessed by MitoSOX Red staining. The data showed that mitochondrial ROS were obviously increased in *Zdhhc13*-deficient hepatocytes than in WT hepatocytes (Fig. 6c). For the observation of hypermetabolism and heat production of the *Zdhhc13*-deficient mice, we examined the protein expression of the uncoupling protein (UCP). The data show that the UCP1 expression in the hepatocyte of the *Zdhhc13*-deficient mice was higher than in the WT. And the oxidative damage biomarkers assessed by SOD2 and Carboxymethyl Lysine (CML) were all higher in *Zdhhc13*-deficient hepatocytes than in WT hepatocytes (Fig. 6d). Moreover, based on IPA annotation, 18 candidate ZDHHC13 substrates, including LDHA, PRDX1, and NDUFS1, were identified as inhibitors of ROS production^36–38^, and CAT was identified as a ROS scavenger^39^ (Table S6). Collectedly, our data support the mitochondrial dysfunction and oxidative stress in the hepatocytes of *Zdhhc13*-deficient mice. To further investigate the effect of ZDHHC13 on mitochondrial function, we knocked-down *Zdhhc13* expression in Hep1–6 cells using shRNA. *Zdhhc13* mRNA expression was reduced by 10–30% in four *Zdhhc13* shRNA clones (A1, B1, C1 and A5) as compared to expression levels of scrambled control shRNA (A6) (Fig. 7a). The spare respiratory capacity was lower in *Zdhhc13*-knockdown cells than in the scramble control in the presence of 0.5 μM FCCP (Fig. 7b). Silencing of *Zdhhc13* led to lower mitochondrial membrane potential compared with that of the scrambled control (Fig. 7c). As expected, knockdown of *Zdhhc13* also led to the production of more mitochondrial ROS (Fig. 7d). In summary, these data support that knockdown of *Zdhhc13* disrupts mitochondrial function in Hep 1–6 cells.

## Discussion

In this study, we observed *Zdhhc13*-deficient mice to exhibit hypermetabolism and defective lipid metabolism and attempted to elucidate the molecular mechanisms underlying these disease phenotypes. We applied an alkylating RAC-based label-free quantitative proteomic strategy to systematically identify S-palmitoylated candidate ZDHHC13 substrates in the liver. With good blocking efficiency and an irreversible chemical reaction to reduce S-palmitoylation false positives, a total of 2,190 unique S-palmitoylated peptides corresponding to 883 proteins were identified and 1905 unambiguous S-palmitoylation sites were determined. Among the identified S-palmitoylated proteins, 311 were novel palmitoylated proteins with unambiguous palmitoylation sites that had not been observed previously by other proteomics studies. We not only successfully identified more than twice the number of palmitoylated proteins as had been previously determined in acyl-RAC-based palmitoylome study using MMTS blocking^15^, but we also effectively determined precise S-palmitoylation sites. This technical advance helps overcome the challenge presented by the fact that some palmitoylated proteins exhibit relatively low levels of palmitoylation or have multiple cysteine residues in their peptide sequences. In addition, complete blocking also decreases the false positive signal from free cysteines and from protein–protein or protein–reagent disulfide linkages^40^. When compared with the results of two palmitoyl-proteomics studies involving ZDHHC5 and ZDHHC17, our method identified 254 and 105 of the S-palmitoylated proteins found in these earlier studies, respectively. This indicates that different PATs may share the same substrates. Compensatory effects between differentially expressed PATs may regulate the extensive palmitoylation of proteins. In addition, diverse palmitoylation profiles may be due to tissue-specific expression of PATs.

Quantitative proteomics analysis revealed potential candidate substrates of ZDHHC13, as defined by proteins with unambiguous palmitoylation sites where the palmitoylation level was ≥0.5-fold lower in *Zdhhc13*-deficient mutants. Utilizing the peptide alignment strategy for label-free quantitation, we further increased the identification coverage and decreased heterogeneity from individual mice. Integrating multiplex quantitation with TMT10-plex isotopic labelling, the palmitoylation levels of these potential candidate substrates were further examined by normalizing with each protein’s expression level. However, in some cases, decreased palmitoylation can affect protein abundance, as palmitoylation is strongly related to protein stability^3^. Thus, further validation of the palmitoylation-mediated biological functions of candidate substrates should be performed in the future.

*Zdhhc13*-deficient mice present with metabolic abnormalities, including non-thyroidal hypermetabolism and hyperhidrosis. Compared to WT mice, *Zdhhc13*-deficient mice consume more food and water but exhibit poor weight gain. Our MS-based systematic analysis demonstrated that 111 of 307 candidate ZDHHC13 substrates were associated with metabolic disease, including mitochondrial dysfunction. Of these, SDHA, NDUFA7, NDUFV1, NDUFS1, and UQCRC1 are part of the mitochondrial respiratory chain, suggesting that defective S-palmitoylation of such ZDHHC13 substrates may contribute to mitochondrial dysfunction. While the ZDHHC13 enzyme is known to be Golgi-localized, we found that many of its substrates (examined in Fig. 4) are nuclear encoded and localized in the mitochondria. We suspect that many of these proteins are palmitoylated in the cytoplasm by ZDHHC13 prior to being imported into the mitochondria. Besides, a sequence motif recognized by Ankyrin repeat domain of ZDHHC13/17 was identified^41^. This may support that ZDHHC13 recognize its substrates by protein sequence rather than a three dimensional protein structure. Notably, palmitoylation was shown to influence the translocation of some proteins, including IRGM1 and BAX, to mitochondria^42, 43^. Moreover, a confirmed mitochondrial palmitoylated protein, CPS1^44^, was also identified as a ZDHHC13 substrate in our study. This suggests that the localization of palmitoylated proteins to mitochondria is a naturally occurring cellular phenomenon. Additionally, high mitochondrial ROS levels in *Zdhhc13*-deficient hepatocyte and *Zdhhc13*-knockdown cells may result from inefficient transfer of electrons across the electron transport chain or abnormal removal of ROS. Indeed, 18 candidate ZDHHC13 substrates function involved in ROS production (Table S6). In humans, mitochondrial dysfunction underlies a clinical phenotype of non-thyroidal hypermetabolism called Luft’s syndrome^45^. Patients with this syndrome exhibit profuse perspiration and lose weight despite adequate food and water intake; at the cellular level, their mitochondria respire profusely and give off excess energy as heat. The causative gene for Luft’s syndrome is still unknown. It would be interesting to investigate whether Luft’s syndrome in humans is caused by a defect in one of these ZDHHC13 substrates.

*Zdhhc13-*deficient mice in this study exhibited mitochondrial dysfunction similar to that in mice carrying a proofreading-deficient mitochondrial DNA polymerase^46^ or *Mcat*-knockout mice^33^. MCAT, a ZDHHC13 substrate identified in this study (Fig. 5), is functionally involved in the *de novo* synthesis of fatty acyl and lipoyl moieties in mitochondria^47^. The non-palmitoylated mutant form of MCAT (C104G) did not exhibit an altered cellular localization (Fig. S2). This suggests that eliminating the palmitoylation of MCAT may disturb its enzyme activity rather than influence its cellular localization.

*Zdhhc13-*deficient mice also exhibited lipid abnormalities with low blood triglyceride and cholesterol levels and decreased lipid accumulation in the liver (Fig. 1a,b). Our proteomics results identified several ZDHHC13 candidate substrates that were functionally involved in lipid metabolism, such as FASN, which is involved in lipogenesis, and ACSL1, which is involved in β-oxidation. In these proteins, palmitoylation may provide a membrane anchor for preferred localizations, such as the inner mitochondrial membrane, or may influence their binding affinity with substrates. Since we identified many enzymes involved in lipid metabolism, it is possible that these enzymes form covalent acyl-enzyme intermediates during catalysis. To eliminate this possibility, we confirmed the palmitoylation of three proteins, ACAA2, MCAT, and CTNND1, by MLCC and demonstrated that they are indeed palmitoylated proteins (Fig. S1). In addition, CTNND1, a specific ZDHHC13 substrate confirmed in this work, is a well-known anti-adipogenic factor^48^. It has been reported that cytoplasmic CTNND1 inhibits GLUT-4 membrane trafficking by regulation of RHO and RAC^34^. As the palmitoylated site C618 is located in the RHO binding domain of CTNND1^49^, it is possible that loss of palmitoylation at C618 may disturb the binding of CTNND1 to RHO, thereby inhibiting lipid accumulation. It would be interesting to know whether palmitoylation of CTNND1 plays a role in inhibiting lipid accumulation, and this should be investigated in the future.

In summary, this is the first study to demonstrate that defects in ZDHHC13, a PAT, leads to phenotypes in mice, including non-thyroidal hypermetabolism, mitochondrial dysfunction, and abnormal lipid metabolism. Here, we used a label-free quantitative proteomics strategy to comprehensively study the liver S-palmitoylome, allowing identification of candidate ZDHHC13 substrates and their functions. Silencing of *Zdhhc13* confirmed that ZDHHC13 is important for mitochondrial function. This study not only revealed the S-palmitoylome for discovery of ZDHHC13 substrates but also shed light on the roles of ZDHHC13 and S-palmitoylation in mitochondrial dysfunction and hypermetabolism. We anticipate that this study will be useful in developing therapeutic strategies for diseases associated with mitochondrial dysfunction and hypermetabolism in the future.

## Materials and Methods

### Mice and genotyping and cell lines

The *Zdhhc13-*deficient mice were generated by ENU mutagenesis as described previously^31^. Genotype was analysed by sequencing tail genomic DNA. All the animals and protocols (IACUC number: 11-05-187) used in this study were approved by the Institutional Animal Care and Utilization Committee of Academia Sinica. All methods were performed in accordance with the relevant guidelines and regulations. HEK-293T, Hep1-6, 3T3-L1 and MCF-7 cells were purchased and cultured as described by the American Tissue Culture Collection (ATCC).

### Metabolic rate measurement

The metabolic performance was measured using a PhenoMaster System (TSE Lab Master System, Homburg, Germany). Six-week-old mice (n = 8) were placed in the PhenoMaster open-circuit indirect calorimeter at room temperature (22 °C–24 °C). Mice were allowed to acclimatize in the chambers for at least 24 h. Food and water were provided ad libitum and measured by the built-in automated instruments. VO_2_, VCO_2_, and heat production were measured for the following 48 h.

### Extraction of membrane proteins from mice liver

Liver tissue samples from 6-week-old mice were homogenized in LB buffer (150 mM NaCl, 50 mM Tris, 5 mM EDTA; pH 7.2) with 10 mM N-ethylmaleimide (NEM, Sigma-Aldrich), 2X protease inhibitor (PI, Roche), and 2 mM PMSF for 2 min at 24,000 rpm in a Polytron PT3000 homogenizer. Tissues were further sonicated using a Branson Sonifier 150 with 10 cycles of 1 s ON and 2 s OFF. The total membrane fraction was collected by high speed centrifugation (200,000 × *g*, 30 min, 4 °C) and was resuspended in 3 mL LB buffer with 1.7% Triton X-100, 10 mM NEM, 1X PI, and 1 mM PMSF for 1 h at 4 °C with end to end rotation. Particles were removed by low-speed centrifugation (250 × *g*, 5 min, 4 °C). The membrane pellet was precipitated by chloroform-methanol precipitation and incubated with 4SB buffer (4% SDS, 50 mM NEM, 50 mM Tris, 5 min EDTA; pH 7.2) at 37 °C until it dissolved. The membrane proteins were applied in MS analysis and western blotting.

### Alkylating Resin-assisted capture (RAC), on-resin digestion, and peptide elution

Detection of S-palmitoylation was performed using the alkylating RAC method^50^ with minor modifications as our previous strategy^40^. In brief, the concentration of NEM-blocked membrane proteins from liver tissues of WT or *Zdhhc13-*deficient mice was adjusted to 2 mg/mL and reacted with final 5 mM TCEP (Sigma-Aldrich) at 37 °C for 30 min to reduce all disulfide bonds. Then the samples were reacted with final 200 mM iodoacetamide (IAM, Sigma-Aldrich) in blocking buffer (20 mM HEPES buffer, pH 7.7, plus 5% (w/v) SDS) at 37 °C for 2 h in the dark to block all of the reduced cysteine residues. After acetone precipitation to remove excess IAM, the mixture was centrifuged at 16000 × *g* for 20 min. The protein pellet was then washed three times with 95% ice-cold ethanol and resuspended in 20 mM HEPES (pH 7.2)/1 mM EDTA buffer/1% SDS. The protein concentration was determined using the BCA Protein Assay Kit (Pierce) and adjusted to 2 mg/ml. The S-palmitoyl groups on proteins were reduced with or without final 0.7 M hydroxylamine (HA, pH 7.2, Sigma, St. Louis, MO) at 37 °C for 2 h to reduce the thioester linkages. After removing excess HA by acetone precipitation and washed three times with 95% ice cold ethanol, the protein pellets were resuspended in 20 mM HEPES (pH 7.2)/1 mM EDTA buffer/1% SDS. In particular, equal amounts of proteins (1.5 mg for mass spectrometry and 150 μg for immunoblot experiments) were harvested and the concentration was adjusted to 1–2 mg/mL. For quantitation by MS analysis, 1 μg BSA protein was added into each sample as the internal standard protein. Then, 300 μl (5:1, proteins: sepharose, w/v) of activated thiol sepharose 4B (GE Healthcare) pre-washing three times with degas PBS was reacted with each sample at 30 °C for 1 h with frequent vortexing. After washing three times by PBS and removing the buffer, the capture proteins on sepharoses were resuspended by 25 mM TEABC (Sigma-Aldrich) and digested with 25 μg trypsin at 37 °C for 16 h with frequent vortexing. The supernatant of digested samples was removed and the captured sepharoses were washed three times by PBS. The captured peptides on sepharoses were resuspended in 25 mM TEABC, eluted by final 5 mM TCEP at 37 °C for 30 min, and dried completely under vacuum. The eluted peptides were desalted by C18 Zip-tip^TM^ (Millipore) and subjected to downstream MS analysis. Triplicate biological experiments and LC-MS/MS analyses were performed on each sample to obtain confident identification and quantitation.

### TMT10-plex labelling of membrane proteins

Membrane proteins from each sample after reduction and alkylating were precipitated by ice acetone and resuspended by 6 M urea in 50 mM HEPES (pH8.2). Protein extracts (10 μg for each) were then diluted to 1 M urea with 25 mM TEABC, and 0.5 μg of trypsin was added and incubated overnight at 37 °C. After stopped by acidification with 0.1% TFA (v/v), peptides were dried and resuspended by 20 μl of 100 mM TEABC. Ten micrograms of peptides from each sample was labeled with TMT reagent. TMT10-plex reagents (0.8 mg) were dissolved in anhydrous acetonitrile (80 μL), of which 8 μL was added into the peptides making final acetonitrile concentration of approximately 30% (v/v). The labelling reaction proceeded for 1 h at room temperature and then was quenched with 1.6 μl of 5% HA to a final concentration of 0.3% (v/v). The TMT-labeled samples were mixed equally and vacuum centrifuged to near dryness. Finally, 20 μg of TMT10-labeled peptide mixtures were fractionated by high pH reverse-phase stage-tip to 8 fractions and dried.

### Mass Spectrometry and data analysis

Eluted palmitoylated peptides mixtures from alkylating RAC and TMT10-labeled peptide mixtures from membrane subfraction were analysed by LTQ-Orbitrap XL and LTQ-Orbitrap Fusion MS, respectively. All MS raw data were processed with Proteome Discover version 2.1 (Thermo Fisher Scientific). For label-free quantification of palmitoylated peptides, data analysis was performed using IDEAL-Q software [34]. Membrane proteomic quantitation was performed by Proteome Discover version 2.1. Full experimental procedures are available in the supplementary data.

### Protein annotation

For subcellular localization, molecular function annotations, and information linked to diseases, all identified proteins were analysed using the Ingenuity Pathway Analysis (IPA, http://www.ingenuity.com) Knowledge Base and the Gene Ontology (GO) consortium. The potential protein-protein interactions and networks of the quantified S-palmitoylated proteins were further annotated by literature mining and IPA analysis.

### Palmitoylation assay

cDNA of WT (ZDWT) and *Zdhhc13*-mutant Flag (ZDK) expression vector, without the DHHC domain which mimic the Zdhhc13 mutation in our mouse model were subcloned into p3XFLAG-CMV14 vectors (Sigma-Aldrich). Mouse *Mcat*, *Ctnnd1 and Acaa2* were subcloned into pcDNA4/myc-His expression vectors (Invitrogen). *Mcat* or *Ctnnd1* expression vectors were subsequently co-transfected with Flag vector, ZDHHC13-Flag-WT (ZDWT) or ZDHHC13-mutant-Flag (ZDK) in HEK-293T cells by using Lipofectamine 2000. After transfection for 48 h, cells were harvested and lysed using lysis buffer (1X RIPA, 1X PI, 1 mM PMSF, 50 mM NEM). The total cell lysate (500 μg) was prepared as an alkylating RAC sample. The Sepharose-captured proteins were eluted using 5 mM TCEP at 37 °C for 30 min and analysed using western blotting.

### Measurement of O_2_ consumption rate

Cells was seeded onto an XF 96-well microplate at a density of 5000 cells/well in hepatocyte or 10000 cells/well in Hep1–6 cells. Cells were incubated in DMEM for 1 h and in DMEM without bicarbonate for 30 min. The microplate was loaded into a XF-96 Extracellular Flux Analyzer (Seahorse Bioscience) following manufacturer’s instructions. Oligomycin, FCCP and Rotenone (Sigma-Aldrich) were injected into the medium sequentially to detect O_2_ consumption rate of cells. All experiments were performed at 37 °C.

The informations about Blood Chemistry, Detection of lipid accumulation by Oil red O stain, Liquid Chromatography and Tandem Mass Spectrometry, Mass Spectrometry Raw data analysis, Label-free quantitation of palmitoylated peptides, Western blotting, *Zdhhc13*-knockdown in Hep1–6 and 3T3-L1 cells, JC1 staining, Measurement of mitochondrial reactive oxygen species (ROS), Metabolic labelling combined with click chemistry and Immunofluorescence assay were addressed in supplementary information.

## Electronic supplementary material

Figure S1

Figure S2

Supplementary table 1, Supplementary table 2, Supplementary table 3, Supplementary table 4, Supplementary table 5, Supplementary table 6

Supplementary Information
