# Supplementary figures and images for: Role of S-Palmitoylation by ZDHHC13 in Mitochondrial function and Metabolism in Liver
Source: Sci Rep. 2017 May 19;7:2182. doi: 10.1038/s41598-017-02159-4 (PMC5438363; doi:10.1038/s41598-017-02159-4)

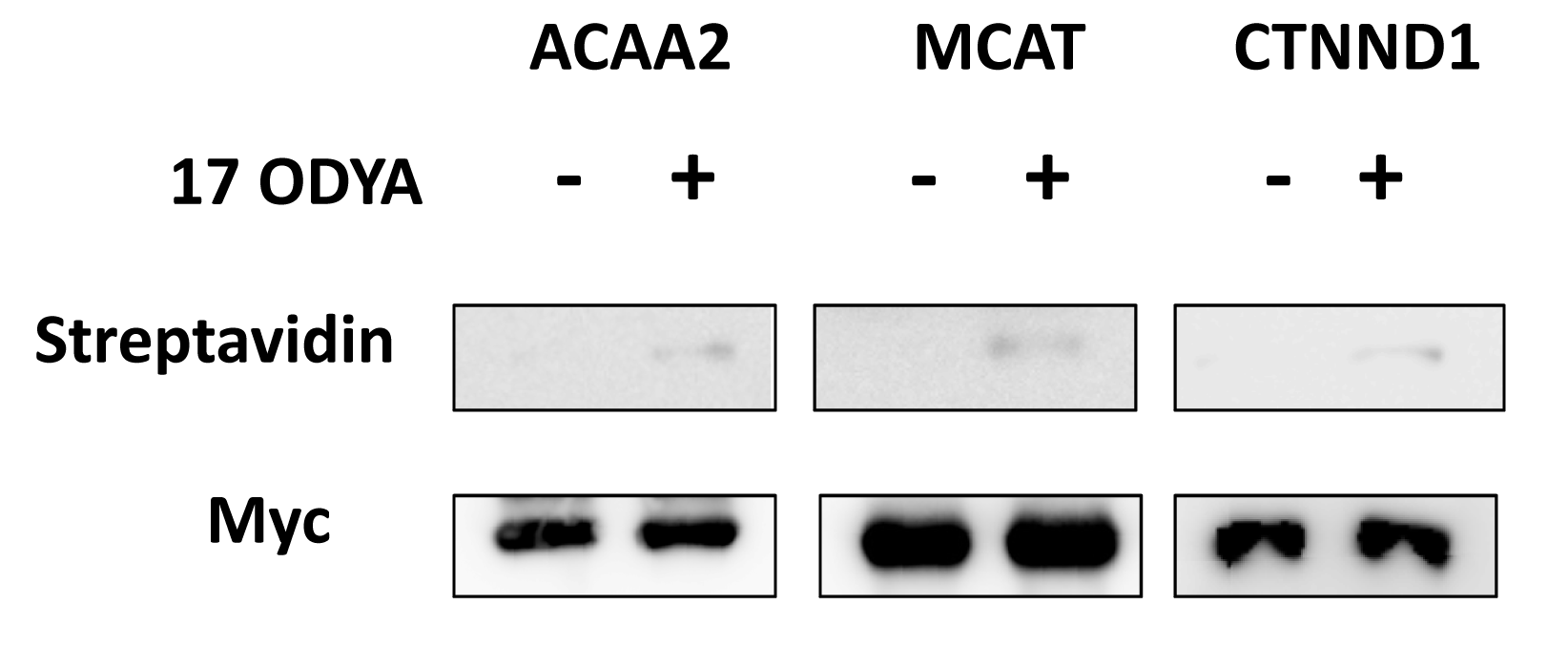

Supplement: Supplementary file 1 — Figure S1 [file 41598_2017_2159_MOESM1_ESM.tif]

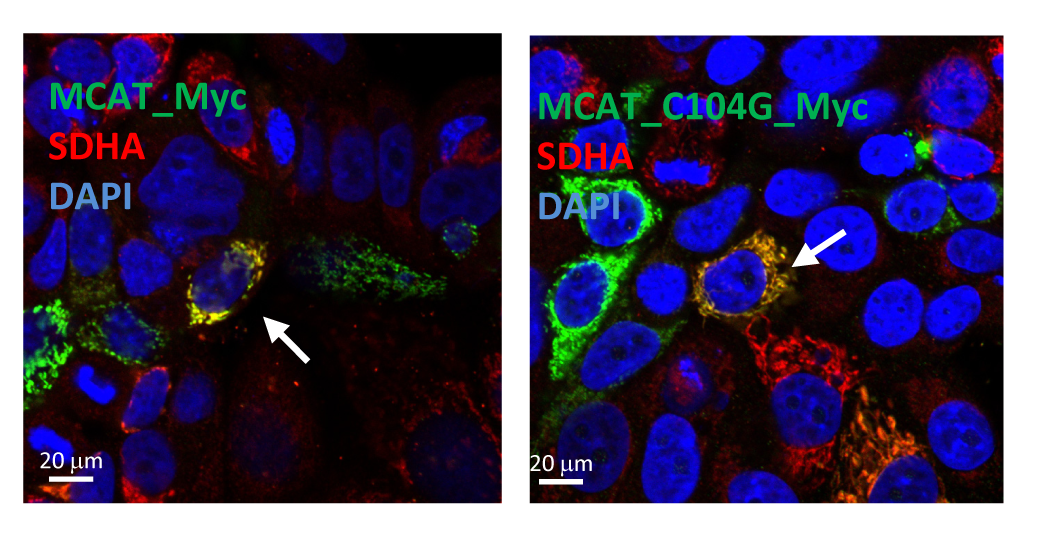

Supplement: Supplementary file 2 — Figure S2 [file 41598_2017_2159_MOESM2_ESM.tif]
