# Supplementary material for: Role of S-Palmitoylation by ZDHHC13 in Mitochondrial function and Metabolism in Liver
Source: Sci Rep. 2017 May 19;7:2182. doi: 10.1038/s41598-017-02159-4 (PMC5438363; doi:10.1038/s41598-017-02159-4)
Supplement: Supplementary file 4 — Supplementary Information [file 41598_2017_2159_MOESM4_ESM.doc]

**Supplementary Information**

**Role of S-Palmitoylation by ZDHHC13 in Mitochondrial function and Metabolism in Liver**

Li-Fen Shen^1^, Yi-Ju Chen^2^, Kai-Ming Liu^1^, Amir N. Saleem Haddad^3^, I-Wen Song^1^, Hsiao-Yuh Roan^1^, Li-Ying Chen^1^, Jeffrey J. Y. Yen^1^, Yu-Ju Chen^2^, Jer-Yuarn Wu^1^, Yuan-Tsong Chen^1,4*^

^1^Institute of Biomedical Sciences, Academia Sinica, Taipei, Taiwan

^2^Institute of Chemistry, Academia Sinica, Taipei, Taiwan

^3^Department of Biology, University of Toronto Mississauga, Mississauga, Ontario, Canada

^4^Department of Pediatrics, Duke University Medical Center, Durham, North Carolina, United States of America

**Supplementary methods**

**Blood Chemistry**

Blood samples were obtained through an incision of the tail artery. Serum was analyzed using the FUJI DRI-CHEM SYSTEM 3500s (Fuji Photo Film Co. Ltd.) for measurement of aspartate aminotransferase (AST; U/l), alanine aminotransferase (ALT; U/l), triglyceride (TG; mg/dl), total cholesterol (TCHO; mg/dl), high-density lipoprotein cholesterol (HDL-C; mg/dl).(n=12). Serum levels of T3 and T4 were detected by T3/T4 ELISA kit (CALBIOTECH) according to the manufacturer’s protocols (n=9).

**Detection of lipid accumulation by Oil red O stain**

Liver samples were immersed in 30% sucrose and frozen in OCT compound (Tissue-Tek). The samples were cut into 10-μm sections and air-dried on slides at room temperature for 30 min. The liver sections were fixed with 4% paraformaldehyde for 1 h, stained with fresh Oil red O for 6 min, and rinsed in 50% isopropanol for few seconds to remove the excess stain. The slides were then viewed under a light microscope. (n=3)

**Liquid Chromatography and Tandem Mass Spectrometry**

NanoLC−nanoESi-MS/MS analysis was performed on a nanoAcquity system (Waters, Milford, MA) connected to an LTQ-Orbitrap Fusion and XL hybrid mass spectrometer (Thermo Fisher Scientific, Bremen, Germany) equipped with a nanospray interface (Proxeon, Odense, Denmark). For label-free quantitation by LTQ-Orbitrap XL MS analysis, peptide mixtures were loaded onto a 75 μm ID, 25 cm length C18 BEH column (Waters, Milford, MA) packed with 1.7 μm particles with a pore with of 130 Å and were separated using a segmented gradient in 148 min from 8% to 45% solvent B (acetonitrile with 0.1% formic acid) at a flow rate of 300 nl/min and a column temperature of 35°C. Solvent A was 0.1% formic acid in water. The mass spectrometer was operated in the data-dependant mode. Briefly, survey full scan MS spectra were acquired in the orbitrap (m/z 350–1600) with the resolution set to 60,000 at m/z 400 and automatic gain control (AGC) target at 106. The 10 most intense ions were sequentially isolated for CID MS/MS fragmentation and detection in the linear ion trap (AGC target at 7000) with previously selected ions dynamically excluded for 90 s. Ions with singly and unrecognized charge state were also excluded. All the measurements in the orbitrap were performed with the lock mass option for internal calibration. For TMT10-plex identification by LTQ-Orbitrap Fusion MS analysis, peptides were loaded onto a 75 μm ID, 25 cm length C18 BEH column (Waters, Milford, MA) packed with 1.7 μm particles with a pore of 130 Å and were separated using a segmented gradient for 130-min segmented gradient with the following mobile phases: water with 0.1% formic acid (buffer A) and 2% to 80% acetonitrile with 0.1% formic acid (buffer B) at 500 nL/min flow rate. Briefly, survey scans of peptide precursors from 300 to 1650 m/z were performed at 120K resolution with a 3 x 10^5^ ion count target detected by orbitrap. Tandem MS was performed by isolation window at 1.4 Da with the quadrupole, and used of step HCD fragmentation, where a given precursor is fragmented with triple HCD collision energies and scanned out in a mass spectrum. The normalized collision energy was set to 36, and HCD fragmentation was detected in orbitrap at a resolution setting of 60K. The MS2 ion count target was set to 1.5 x 10^5^ and the max injection time was 50 ms. Only those precursors with charge state 2–6 were sampled for MS2. The instrument was run in top speed mode with 3 s cycles; the dynamic exclusion duration was set to 40 s with a 10 ppm tolerance around the selected precursor and its isotopes.

**Mass Spectrometry Raw data analysis**

All LC-MS/MS raw data were processed with Proteome Discover version 2.1 (Thermo Fisher Scientific), and peptides were searched against SwissProt database (version 2016_05, *Mus musculus*, 16,840 sequences) with constraints including peptide missed cleavage sites lower than two, precursor ion mass tolerance 10 ppm. For label-free identification with LTQ-Orbitrap XL MS analysis with CID, fragment ion tolerance 0.6 Da. Carbamidomethyl (C), Nethylmaleimide (C), deamidation (NQ), and oxidation (M) were selected as variable modifications. False discovery rates (FDRs) were calculated by target-decoy strategy with Mascot Percolator. All peptide hits were filtered with a 1% FDR cutoff. For TMT10-plex labeling with LTQ-Orbitrap Fusion MS analysis, Proteome Discover version 2.1 was integrated Mascot, SEQUEST, and MSAmanda to process the protein identification. Fragment ion tolerance was set as 0.05 Da. TMT10plex (KR and N-term), Carbamidomethyl (C), Nethylmaleimide (C), deamidation (NQ), and oxidation (M) were selected as variable modifications. False discovery rates (FDRs) were calculated by target-decoy strategy with Percolator. All peptide hits were filtered with a 1% FDR cutoff. Reporter ions quantifier was calculated by Proteome Discoverer with the integration tolerance of 0.02 Da.

**Label-free quantitation of palmitoylated peptides**

For label-free quantification of palmitoylated peptides, data analysis was performed using IDEAL-Q software [^1^](#_ENREF_1). The raw data files from LTQ-Orbitrap XL MS analysis were converted to mzXML format by MSConvert (ProteoWizard 3.0). After the Mascot database search, the confidently identified peptides (p < 0.05) from each LC-MS/MS run were exported in eXtensive Markup Language (XML) data format. IDEAL-Q was used to quantitatively analyze the xml files and the corresponding mzXML files. IDEAL-Q performed the ID-based elution time prediction using a fragmental regression (IDEAL) algorithm to predict the retention time of the peptide in the current run. Based on the retention times of the identified peptides, unidentified peptides can be detected and aligned by performing peak cluster detection near the peptide m/z (< 0.1 Da) and predicted elution time (< 1.5 min). Integrating the identification results from all LC-MS/MS runs into a global peptide information list, including different sequences, charge states, and modification sites, these unidentified peptides, termed assigned peptides, were detected and aligned. To ensure quantitation accuracy, all peptide peaks were validated by the following criteria: signal-to-noise (S/N) ratio > 3, correct charge state, and correct isotope pattern. To calculate the relative peptide abundance, the tool uses the area under the curve of extracted ion chromatography (XIC), and the calculated XIC area is further normalized by the XIC area of the internal standard peptide containing cysteine residue. The fold change in a given peptide was calculated by the log_2_-transformed ratio of the normalized peptide abundance between different samples. This tool also calculates the mean of all log_2_-transformed peptide ratios as the normalization coefficient to perform central tendency normalization to reduce systematic biases. Ratios in the region outside the mean ±2 SD (95% confidence interval, p < 0.05) were considered differentially expressed. The XIC results of the differentially expressed S-palmitoylated peptides were further validated manually. The relative quantitative ratio was determined by the sample abundance of the corresponding peptide.

**Western blotting**

Tissues were processed with the same procedure of RAC sample preparation. The protein extract was separated on SDS-PAGE and transferred to Nitrocellulose Transfer membrane (Whatman). After blocking with 5% milk, primary antibodies were used for WB detection. Primary antibodies included rabbit anti-Sdha, rabbit anti-FASN (Abcam), rabbit anti-NDUFV1, rabbit anti-MCAT (Santa Cruz), rabbit anti-NDUFS1, rabbit anti-UQCRC1, rabbit anti-ACAA1, rabbit anti-ACAA2, rabbit anti-GAPDH (Bioworld), rabbit UCP1, rabbit SOD2 (Gene Tex), rabbit anti-CML (Acris), mouse anti-CTNND1 (Thermo), mouse anti-FLAG (Sigma-Aldrich), and mouse-anti-Myc (Millipore), mouse anti-Tubulin (Abcam). HRP-conjugated goat anti-mouse and goat anti-rabbit secondary antibodies (KPL) were applied to the primary antibodies. Streptavidin conjugated HRP (GeneTex) was applied to detect biotin. The protein bands were visualized by enhanced chemiluminescence development (PerkinElmer).

**JC-1 staining**

In accordance with JC-1 guidelines (Molecular Probes), up to 1 × 10^6^ cells/mL were used for the assay. 3T3-L1 cells were collected by centrifugation in 1,000 x *g* 5 min, and re-suspended in PBS. Cells were incubated with 2 µM JC-1 at 37°C for 30 min, washed twice in PBS, and re-suspended in 1 mL PBS. Mitochondrial membrane potentials (ΔΨm) were estimated by determining relative amounts of dual emissions from JC-1 monomers or aggregates by using a BD FACSCanto flow cytometry system. The red/green fluorescence was calculated using the FACSDivia.

**Measurement of mitochondrial reactive oxygen species (ROS)**

Mitochondrial ROS level was monitored by using the MitoSOX Red (Molecular Probes). Up to 1 × 10^6^ cells/mL cells were collected, by centrifugation in 1,000 x *g* 5 min, and re-suspended in HBSS buffer. Cells were incubated with 5μM MitoSOX Red for 1 hour in the dark. Then, cells were washed twice with HBSS buffer, re-suspended in culture medium. Thereafter, cells were analyzed using a flow cytometer (FACSCanto, BD).

***Zdhhc13*-knockdown in Hep1-6 and 3T3-L1 cells**

The lentivirus containing *Zdhhc13*-targeting small hairpin RNA (shRNA) was prepared by the National RNAi Core Facility (Taipei, Taiwan). The shRNAs targeting *Zdhhc13* were A1, 5’-CCCAAGAACATGAGTTACTTT-3’ (TRCN0000081753); B1, 5’- ATTCAGCTACCACGGTCAAAG-3’(TRCN0000433751); C1, 5’- ACCTAATAGTCACTGATTAT-3’ (TRCN0000429407); and A5, 5’- CCAACAAACAGTTTCGGCTTT-3’ (TRCN0000081757). One shRNA was used as the scramble control: A6, 5’-GCTGAGTACTTCGAAATGTCC-3’ (TRCN0000072264). Cells were infected with lentivirus (MOI = 4) in DMEM containing 8 μg/mL polybrene (Sigma-Aldrich). At 24 h post-infection, cells were incubated in fresh DMEM with 4 μg/mL Puromycin (Sigma-Aldrich) for 7 days. *Zdhhc13* mRNA expression was evaluated by qPCR using the Power SYBR green PCR Master Mix (Applied Biosystems, Foster City, CA, USA) and an ABI PRISM 7700 Sequence Detection System. Gene expression was normalized to that of *Gapdh* for the fold change calculation. The primers used were as follows: *Zdhhc13*, F-5’- CGGAGGAAGAGAGGAAAGTG-3’, R-5’-TGGAAAGGGAAAGCAAGAAG-3’; and *Gapdh*, F-5’-CCAGAACATCATCCCTGCAT, R- 5’-GTTCAGCTCTGGGATGACCTT.

**Metabolic Labeling Combined with Click Chemistry**

Transfected HEK293 cells were cultured in DMEM with 10% FBS for 24 h. Cells were then incubated in DMEM containing 5% dialyzed FBS and 75 μM 17-ODYA (Cayman Chemical) or DMSO for 12h. Cells were harvested and performed the membrane protein extraction. Membrane proteins were immunoprecipitated with anti-Myc and protein G agarose (GE Healthcare) for overnight. The immunoprecipitates were washed 3 times with LB buffer containing 0.5 % TritonX-100 and eluted by 46.5 μl of 50 mM HEPES pH7.4, 150 mM NaCl, 2 % SDS for 30 min. Eluates were performed click reaction by addition 100 μM biotin-azide (Thermo Fisher Scientific), 1 mM tris(2-carboxyethyl)phosphine (TCEP), 100 μM tris[(1-benzyl-1H-1,2,3-triazol-4-yl)methyl]amine (TBTA, Sigma-Aldrich ), and 1 mM CuSO4) for 1h at RT. Samples were then denatured with SDS sample buffer, heated at 80 °C for 5 min and subjected to SDS/PAGE and western blot.

**Immunofluorescence assay**

MCF-7 cells were fixed with 4% paraformaldehyde in PBS and permeabilized with 0.1% Triton X-100 (in PBS) 10 min at room temperature. After blocking with 5% normal goat serum for 1 h, cells were incubated overnight with the following primary antibodies: mouse anti-Myc (1:500; Millipore) and rabbit anti-SDHA (1:100; Abcam). Secondary antibodies used were anti-mouse Alexa Fluor 488 (1:500; Jackson ImmunoResearch), anti-rabbit Alexa Fluor 594 (1:400; Jackson ImmunoResearch). Nuclei were stained with DAPI by using the ProLong Gold Antifade Reagent (Invitrogen). Images were captured using an Axiovert 200M (Zeiss).

**Supplementary Figures and legends**


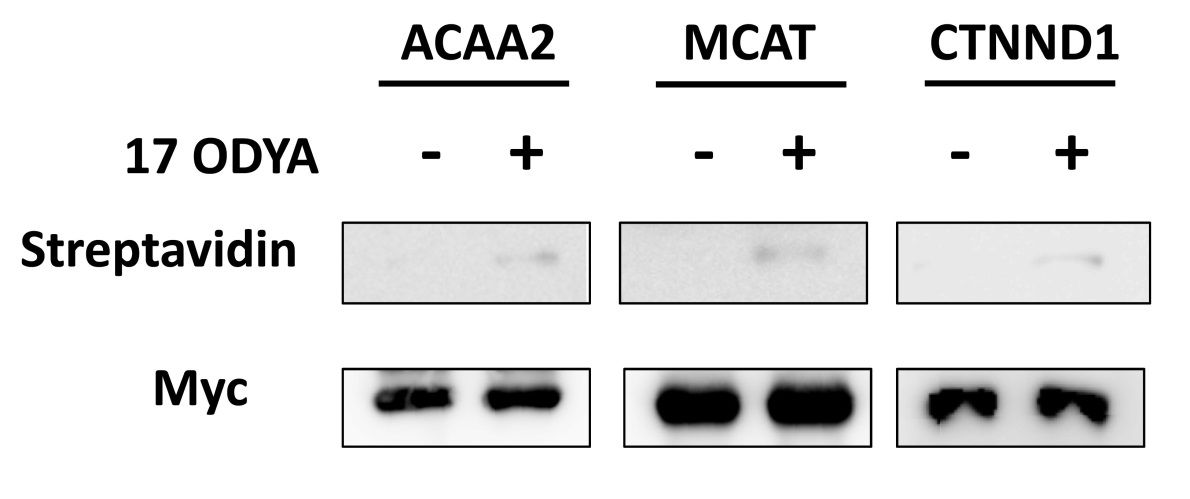


Supplementary Figure 1. **ACAA2, MCAT and CTNND1 are palmitoylated protein**. HEK293 cells transfected with ACAA2_Myc, MCAT_Myc or CTNND1-Myc were incubated in medium containing 75μM 17-ODYA or DMSO for 12 h. Lysates were immunoprecipitated with anti-Myc antibody, the precipitated proteins were eluted and performed click reaction, resolved by SDS–PAGE and immunoblotted with Streptavidin HRP or anti-Myc. This experiment was repeated 3 times.


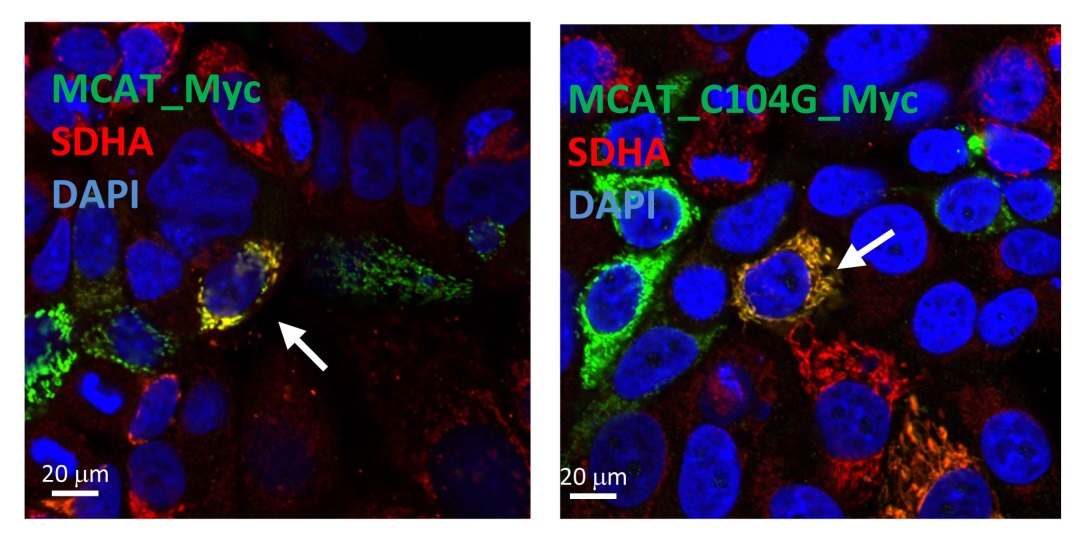


Supplementary Figure 2. **Effect of palmitoylation on the subcellular localization of ZDHHC13 substrate MCAT.** MCF-7 cells were transfected with MCAT_Myc or mutant C104G form. MCAT_Myc distribution was analyzed by Myc primary antibody and visualized with Alexa Fluor 488 (green). Mitochondrial marker of endogenous SDHA was analyzed by anti-SDHA and visualized with Alexa Fluor 594 (red). Nucleus: DAPI (blue). White arrow indicated the colocalized signal of SDHA and MCAT in cell.

**Legends to Supplementary Tables**

Supplementary table 1. Peptide derived from S-palmitoylated proteins identified in WT and *Zdhhc13-*deficient mice liver using alkyl-RAC method. S-palmitoylated proteins are list in line included the uniport number, gene name, protein name, mass to charge ratio (m/z), peptide charge, peptide sequence which the modification cysteine is shown in lower case letter, peptide score, S-palmitoylated modification site, the ratio of K+/WT+ displayed as the abundance of *Zdhhc13-*deficient groups divided by WT groups (“+” indicated the sample treated with Hydroxylamine), the ratio of K+/K and W+/W displayed as the abundance of samples treated with or without Hydroxylamine, and the protein location. (K indicates *Zdhhc13-*deficient mice).

Supplementary Table 2. There are 311 novel S-palmitoylated proteins identified in this study. The proteins are list in line included the uniport number, gene name, protein name, protein mass and mass to charge ratio (m/z).

Supplementary table 3. TMT10-based quantitation of membrane proteome from liver tissues of five pairs of WT and *Zdhhc13-*deficient mice. Total proteins are list in line included the uniport number, description/protein name, coverage, peptide number, peptide spectrum matching (PSMs), number of unique peptides, molecular weight (MW). The ratio of K/WT is displayed as the average intensity of the reporter ions of TMT10-plex. The WT samples are labeled by tag 126-128, and *Zdhhc13-*deficient samples are labeled by tag 129-131. (K indicates *Zdhhc13-*deficient mice).

Supplementary table 4. The remaining 254 proteins corresponding to 400 S-palmitoylation sites (369 peptides) were annotated as potential ZDHHC13 substrates. S-palmitoylated proteins are list in line included the uniport number, gene name, protein name, mass to charge ratio (m/z), peptide charge, peptide sequence which the modification cysteine is shown in lower case letter, peptide score, S-palmitoylated modification site, the ratio of K+/WT+ displayed as the abundance of *Zdhhc13-*deficient groups divided by WT groups (“+” indicated the sample treated with Hydroxylamine), the ratio of K+/K and W+/W displayed as the abundance of samples treated with or without Hydroxylamine.(K indicates *Zdhhc13-*deficient mice).

Supplementary table 5. The potential ZDHHC13 substrates identified from this study were analysis by IPA. Among them, there are 142 proteins involved in metabolism in the category of biological function and diseases or functions annotation.

Supplementary table 6. Based on IPA analysis, there are 18 potential ZDHHC13 substrates which are defined as association with ROS production.

**Supplementary Data References**

1 Tsou, C.-C. *et al.* IDEAL-Q, an automated tool for label-free quantitation analysis using an efficient peptide alignment approach and spectral data validation. *Mol. Cell. Proteomics* **9**, 131-144, doi:10.1074/mcp.M900177-MCP200 (2010).
